# A risk stratification model identifies a high-risk subgroup for connective tissue disease among dermatology patients with positive antinuclear antibody testing: A retrospective cohort study

**DOI:** 10.1016/j.jdin.2026.05.007

**Published:** 2026-05-14

**Authors:** Chang Hun Lee, Yunjung Choi

**Affiliations:** a Research Institute of Clinical Medicine of Jeonbuk National University - Biomedical Research Institute, Jeonbuk National University, Jeonju, South Korea; b Division of Hepatology, Department of Internal Medicine, Jeonbuk National University Medical School, Jeonju, South Korea; c Division of Rheumatology, Department of Internal Medicine, Jeonbuk National University Medical School, Jeonju, South Korea

**Keywords:** antibodies, antinuclear, connective tissue diseases, dermatology, epidemiology, patient referral, risk assessment

Antinuclear antibody (ANA) positivity is frequently encountered in dermatology practice, yet only a minority of patients who are ANA-positive are ultimately found to have connective tissue disease (CTD).^1^, ^2^, ^3^ Nevertheless, a positive ANA result often triggers rheumatology referral regardless of the clinical context.^4^ No validated tool currently exists to inform referral decisions in this population. We sought to develop and internally validate a risk stratification model that identifies patients with ANA-positive dermatology with a clinically meaningful and substantially higher probability of CTD.

In this retrospective study, we reviewed rheumatology-naïve outpatients who underwent dermatologist-ordered ANA testing at a tertiary university hospital between 2013 and 2023 (IRB approval: JNUH 2024-09-007-001). Of 7448 patients tested, 944 (12.7%) were ANA-positive and formed the analytical cohort. CTD was diagnosed by board-certified rheumatologists applying ACR/EULAR classification criteria. A multivariable logistic regression model using clinical and serologic variables available at the time of evaluation was developed with stratified five-fold cross-validation (detailed methods in Supplementary Material, available via Mendeley at https://data.mendeley.com/datasets/s4yv3x2fh4/1).

Fifty-eight of the 944 patients who are ANA-positive (6.1%) received a CTD diagnosis. Baseline characteristics are presented in Table I. Those with CTD tended to be younger (mean 44.2 vs 52.5 years) and were more often female (72.4% vs 65.9%). Higher ANA titers and cutaneous findings classically associated with CTD were observed more frequently in the CTD group (definitions in Supplementary Table I, available via Mendeley at https://data.mendeley.com/datasets/s4yv3x2fh4/1).

**Table I:** Baseline characteristics of ANA-positive dermatology patients by connective tissue disease diagnosis

	ANA-positive (Total) (*N* = 944)	ANA-positive with CTD (*N* = 58)	ANA-positive without CTD (*N* = 886)
Age, mean ± SD	52.0 ± 19.9	44.2 ± 17.0	52.5 ± 19.9
Sex			
Female	626 (66.3%)	42 (72.4%)	584 (65.9%)
Male	318 (33.7%)	16 (27.6%)	302 (34.1%)
Dermatologic diagnosis category (grouped)			
Eczematous dermatitis	220 (23.3%)	12 (20.7%)	208 (23.5%)
Urticarial/hypersensitivity	111 (11.8%)	6 (10.3%)	105 (11.9%)
Papulosquamous disorders	126 (13.3%)	10 (17.2%)	116 (13.1%)
CTD-like skin manifestations	11 (1.2%)	3 (5.2%)	8 (0.9%)
Others∗	476 (50.4%)	27 (46.6%)	449 (50.7%)
ANA titer category			
Low (≤1:160)	426 (45.1%)	17 (29.3%)	409 (46.2%)
Intermediate (1:320–1:640)	90 (9.5%)	21 (36.2%)	69 (7.8%)
High (≥1:1280)	38 (4.0%)	15 (25.9%)	23 (2.6%)
Missing† (pattern-only or not reported)	390 (41.3%)	5 (8.6%)	385 (43.5%)

Values are presented as mean ± standard deviation or *n* (%), as appropriate.

Baseline characteristics are presented descriptively without formal statistical testing.

∗ Others include vasculitic/purpuric disorders, hair and pigmentary disorders, infections/reactive dermatoses, benign neoplasms/non-inflammatory conditions, and unclassified diagnoses.

† Missing indicates cases in which ANA titers were not reported.

The model showed good discriminative ability (AUROC, 0.80). When patients were divided into tertiles of predicted probability, CTD prevalence rose progressively: 1.9% (6/315) in the low-risk group, 2.9% (9/314) in the intermediate-risk group, and 13.7% (43/315) in the high-risk group (Fig 1)-a sevenfold gradient across strata.

**Fig 1:**
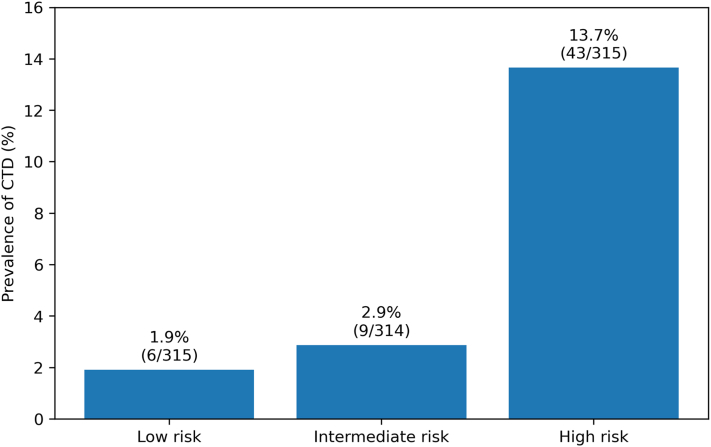
Connective tissue disease risk stratification among dermatology patients with positive antinuclear antibody testing. Prevalence of CTD across low-, intermediate-, and high-risk groups defined by tertiles of predicted probability derived from the internally validated multivariable model. CTD prevalence increased in a stepwise manner across risk strata, from 1.9% (6/315) in the low-risk group to 2.9% (9/314) in the intermediate-risk group, and 13.7% (43/315) in the high-risk group. Patients classified as high risk exhibited sevenfold higher prevalence of CTD compared with those in the low-risk group, demonstrating clinically meaningful risk enrichment despite modest differences in global discrimination metrics.

The absolute difference in CTD probability-from roughly 2% in the lowest tertile to nearly 14% in the highest-is of potential clinical relevance. In practice, such stratification could distinguish patients who warrant early rheumatologic evaluation from those in whom referral may reasonably be deferred, thereby reducing low-yield consultations.

This study is limited by its single-center retrospective design and the small number of CTD events; external validation in independent cohorts is needed. Nonetheless, risk stratification based on routinely available clinical data may provide a practical approach for directing rheumatology referral toward patients with ANA-positive dermatology most likely to harbor underlying CTD.

### Declaration of Generative AI and AI-assisted technologies in the writing process

During the preparation of this work, the authors used ChatGPT (OpenAI, USA) and Claude (Anthropic, USA) to assist with language editing and improving the readability of the manuscript. All AI-assisted content was carefully reviewed, verified, and revised by the authors, who take full responsibility for the integrity and accuracy of the final publication.

## Conflicts of interest

None disclosed.
